# sBGC-hm: an atlas of secondary metabolite biosynthetic gene clusters from the human gut microbiome

**DOI:** 10.1093/bioinformatics/btad131

**Published:** 2023-03-17

**Authors:** Huixi Zou, Tianli Sun, Bangqun Jin, Shengqin Wang

**Affiliations:** National and Local Joint Engineering Research Center of Ecological Treatment Technology for Urban Water Pollution, Wenzhou University, Wenzhou 325035, China; Zhejiang Provincial Key Laboratory for Subtropical Water Environment and Marine Biological Resources Protection, Wenzhou University, Wenzhou 325035, China; College of Life and Environmental Science, Wenzhou University, Wenzhou 325035, China; National and Local Joint Engineering Research Center of Ecological Treatment Technology for Urban Water Pollution, Wenzhou University, Wenzhou 325035, China; Zhejiang Provincial Key Laboratory for Subtropical Water Environment and Marine Biological Resources Protection, Wenzhou University, Wenzhou 325035, China; College of Life and Environmental Science, Wenzhou University, Wenzhou 325035, China; National and Local Joint Engineering Research Center of Ecological Treatment Technology for Urban Water Pollution, Wenzhou University, Wenzhou 325035, China; Zhejiang Provincial Key Laboratory for Subtropical Water Environment and Marine Biological Resources Protection, Wenzhou University, Wenzhou 325035, China; College of Life and Environmental Science, Wenzhou University, Wenzhou 325035, China; National and Local Joint Engineering Research Center of Ecological Treatment Technology for Urban Water Pollution, Wenzhou University, Wenzhou 325035, China; Zhejiang Provincial Key Laboratory for Subtropical Water Environment and Marine Biological Resources Protection, Wenzhou University, Wenzhou 325035, China; College of Life and Environmental Science, Wenzhou University, Wenzhou 325035, China

## Abstract

**Summary:**

Microbial secondary metabolites exhibit potential medicinal value. A large number of secondary metabolite biosynthetic gene clusters (BGCs) in the human gut microbiome, which exhibit essential biological activity in microbe–microbe and microbe–host interactions, have not been adequately characterized, making it difficult to prioritize these BGCs for experimental characterization. Here, we present the sBGC-hm, an atlas of secondary metabolite BGCs allows researchers to explore the potential therapeutic benefits of these natural products. One of its key features is the ability to assist in optimizing the BGC structure by utilizing the gene co-occurrence matrix obtained from Human Microbiome Project data. Results are viewable online and can be downloaded as spreadsheets.

**Availability and implementation:**

The database is openly available at https://www.wzubio.com/sbgc. The website is powered by Apache 2 server with PHP and MariaDB.

## 1 Introduction

Secondary metabolites (SMs) are a series of small bioactive molecules with potential medicinal value that is derived from living organisms, with microorganisms serving as a significant resource (Newman and Cragg 2020). For instance, the majority of known antibiotics and biologically active SMs are derived from the Streptomyces (de Lima Procópio et al. 2012), while thousands of SM biosynthetic gene clusters (BGCs) have been identified in other bacteria (Skinnider et al. 2020; Wei et al. 2021). IMG-ABC, the largest BGC database accessible to the public, stores hundreds of thousands of predicted clusters (Palaniappan et al. 2020). As a result of the explosion of a large amount of BGC data, the prioritization of biologically active clusters for experimental characterization became a challenge. Genome mining has identified a subset of human microbial-derived SM BGCs with essential functional activities (Donia et al. 2014). However, there are still insufficient resources available for human-related BGCs.

Recent implementation of a comprehensive genome collection of the human gut microbiome, including both metagenome-assembled genomes (MAGs) and RefSeq genomes (Hiseni et al. 2021), provides material for the detection of human-related BGCs. As a step towards discovering candidate biology activity SM from the gut microbiome, we created a database called sBGC-hum, an atlas of SM BGCs predicted from the sequenced human gut microbiome. For the prioritization of these BGCs for experimental investigation, their associated metadata, such as BGC family, gene abundance, gender differences (Krumsiek et al. 2015), transporter gene (Crits-Christoph et al. 2021), resistance gene (Yan et al. 2020), etc., are also incorporated. Gene co-occurrence information is also a crucial component for enhancing BGC structure prediction (Libis et al. 2019). To obtain this information, we built a gene co-occurrence matrix using the Human Microbiome Project (HMP) data.

## 2 Data retrieval and processing

HumGut was used to download 10 432 883 genome sequences of the human microbiome (Hiseni et al. 2021). Their protein-coding genes were predicted with Prokka v1.14.6 and annotated with Prodigal v2.6.3, using the parameters –addgenes and –addmrna (Hyatt et al. 2010; Seemann, 2014). All genome sequences in GenBank format were processed by the command-line version of antiSMASH v6.1.1 with the following parameters: –taxon bacteria –asf –rre –clusterhmmer –pfam2go (Blin et al. 2021). Then, the putative BGCs were clustered and classified using the platform BiG-SCAPE with default parameters (Navarro-Muñoz et al. 2020).

Using the extracted protein sequences from predicted BGCs, a local database was constructed in order to estimate the gene abundance of these BGCs in metagenomic datasets. HMP was used to fetch 552 Illumina platform-derived metagenomic datasets from 501 individuals, including 254 females and 247 males (Turnbaugh et al. 2007). The DIAMOND program aligned each metagenomic dataset to the local protein database with the following parameters: -e 0.00001 -b 5 -c 1 -k 200. If a single read identified multiple proteins, only the hits with the lowest E-value were retained. However, reads with more than 200 hits were removed to reduce the false positives (Donia et al. 2014). The metagenomic reads from identical individuals were combined. Finally, each individual's gene abundance was normalized as follows:


(1) $$\left(\mathrm{Aligned}\;\mathrm{reads}\;\mathrm{of}\;\mathrm{gene}/\mathrm{Total}\;\mathrm{number}\;\mathrm{of}\;\mathrm{reads}\right)\;*10^{6}$$

## 3 Main features

sBGC-hm provides a user-friendly interface with the following key characteristics:

1. It is the first human gut microbial-derived BGC database to include the most comprehensive set of SM BGCs to date.
2. Keyword and BLAST searches are provided for acquiring target BGCs. Table 1 lists the items available in sBGC-hm with an example.
3. The gene co-occurrence matrix derived from HMP data is given to enhance BGC structure prediction (Supplementary Fig. S1).
4. Additional visualization is employed for the prioritization, such as the abundance of core biosynthetic genes and the gender differences of these genes.

**Table 1.** A description of the data items in sBGC-hm with an example BGC. (Detailed descriptions are available on the website)^a^

Data items	Examples
Cluster	9
Family	FAM_23957
Organism	Bacteroides sp. CAG:545
Taxon ID	1262742
Start	1
End	22 809
Length	22 809
Type	Arylpolyene ^b^
Gene Count	16 ^c^
Core Genes	1
Transporter Genes	0
Resistance Genes	0
Mean	0.2452 ^d^
Gender Differences	0.04305 ^e^
MiBiG	BGC0000838 (8%)
Download	GenBank

a Underline indicates hyperlinks to external information.

b-e The hyperlinks lead to the BGC structure, the gene co-occurrence matrix, the density plot of gene abundance, and the boxplot of the distribution of gene abundance by gender, respectively.

## 4 Statistics

A total of 36 583 BGCs are detected, with ranthipeptide 27.0% (9872) being the most abundant type. There are also 4.4% (1625) NRPSs and 3.6% (1324) PKSs. Firmicutes account for 73.1% (26 727) of these BGCs, followed by Proteobacteria 10.5% (3840) and Bacteroidota 10.0% (3659). *Streptococcus* contributes the highest number of BGCs at the genus level, with a total of 2800 BGCs classified into 354 families. The average number of genes in these BGCs is 11.8, while the majority contain 3–5 genes. The BiG-SCAPE analysis discovered 8004 families, the largest of which contained 186 BGCs. According to the Wilcoxon rank-sum test of gene abundance, 3406 BGCs are enriched for males using the HMP data analysis. Statistics and figures for these BGCs are available on the website's statistics page.

## 5 Discussion

The majority of bacteria in the human microbiome cannot be cultured at this time. The sequenced bacterial genome provides the information necessary to investigate the biosynthetic capability of these microbiomes. Here, the distribution of BGCs in the human gut microbiome was systematically investigated, supporting that BGCs were abundant. The atlas of SM BGCs allows researchers to explore the potential therapeutic benefits of these natural products.

## Supplementary Material

btad131_Supplementary_Data
