# Supplementary material for: sBGC-hm: an atlas of secondary metabolite biosynthetic gene clusters from the human gut microbiome
Source: Bioinformatics. 2023 Mar 17;39(3):btad131. doi: 10.1093/bioinformatics/btad131 (PMC10049752; doi:10.1093/bioinformatics/btad131)
Supplement: btad131_Supplementary_Data [file btad131_supplementary_data.pdf]

The gene co–occurrence matrix in cluster 216 derived from HMP data (Pearson correlations above 0.5 were highlighted).

No.	1	2	3	4	5	6	7	8	9	10	11	12	13	14	15	
1	1	0.98	0.67	0.97	0.46	0.64	0.66	0.25	0.61	0.27	0.46	0.41	0.61	0.36	0.32	1 OKPGIJNI_756164 biosynthetic
2	0.98	1	0.66	0.97	0.46	0.62	0.64	0.23	0.59	0.21	0.44	0.4	0.58	0.33	0.31	2 OKPGIJNI_756165 regulatory
3	0.67	0.66	1	0.69	0.45	0.75	0.77	0.22	0.45	0.26	0.36	0.35	0.6	0.54	0.58	3 OKPGIJNI_756166 biosynthetic–additional
4	0.97	0.97	0.69	1	0.46	0.61	0.65	0.25	0.6	0.25	0.46	0.43	0.61	0.37	0.34	4 OKPGIJNI_756167 other
5	0.46	0.46	0.45	0.46	1	0.37	0.4	0.11	0.3	0.12	0.18	0.18	0.3	0.25	0.3	5 OKPGIJNI_756168 other
6	0.64	0.62	0.75	0.61	0.37	1	0.87	0.14	0.38	0.18	0.38	0.33	0.54	0.46	0.41	6 OKPGIJNI_756169 regulatory
7	0.66	0.64	0.77	0.65	0.4	0.87	1	0.16	0.43	0.21	0.49	0.44	0.6	0.45	0.47	7 OKPGIJNI_756170 other
8	0.25	0.23	0.22	0.25	0.11	0.14	0.16	1	0.68	0.23	0.17	0.14	0.19	0.13	0.07	8 OKPGIJNI_756171 other
9	0.61	0.59	0.45	0.6	0.3	0.38	0.43	0.68	1	0.38	0.32	0.25	0.44	0.25	0.18	9 OKPGIJNI_756172 other
10	0.27	0.21	0.26	0.25	0.12	0.18	0.21	0.23	0.38	1	0.32	0.14	0.23	0.21	0.15	10 OKPGIJNI_756173 other
11	0.46	0.44	0.36	0.46	0.18	0.38	0.49	0.17	0.32	0.32	1	0.74	0.18	0.11	0.17	11 OKPGIJNI_756174 other
12	0.41	0.4	0.35	0.43	0.18	0.33	0.44	0.14	0.25	0.14	0.74	1	0.28	0.21	0.31	12 OKPGIJNI_756175 other
13	0.61	0.58	0.6	0.61	0.3	0.54	0.6	0.19	0.44	0.23	0.18	0.28	1	0.76	0.5	13 OKPGIJNI_756176 other
14	0.36	0.33	0.54	0.37	0.25	0.46	0.45	0.13	0.25	0.21	0.11	0.21	0.76	1	0.44	14 OKPGIJNI_756177 other
15	0.32	0.31	0.58	0.34	0.3	0.41	0.47	0.07	0.18	0.15	0.17	0.31	0.5	0.44	1	15 OKPGIJNI_756178 other

Supplementary Figures S1. An example of a gene co-occurrence matrix shown on the website.
